# Effective small crack detection based on tunnel crack characteristics and an anchor-free convolutional neural network

**DOI:** 10.1038/s41598-024-60454-3

**Published:** 2024-05-06

**Authors:** Li Wang, Chao Tang

**Affiliations:** 1 The Key Laboratory of Urban Security and Disaster Engineering of China Ministry of Education, Beijing University of Technology, Beijing, China; 2 Beijing Urban Construction and Surveying Design Research Institute CO, LTD, Chaoyang, Beijing, China

**Keywords:** Metro tunnel, Anchor-free, Cracks, Optimized YOLOX-x, Civil engineering, Computer science

## Abstract

Tunnel cracks are thin and narrow linear targets, and their pixel proportions in images are usually very low, less than 6%; therefore, a method is needed to better detect small crack targets. In this study, a crack detection method based on crack characteristics and an anchor-free framework is investigated. First, the characteristics of cracks are analyzed to obtain the real crack texture, interference noise texture, and targets appearing near each crack as the context information for the model to filter and remove noise. We discuss the crack detection performance of anchor-based and anchor-free algorithms. Then, an optimized anchor-free algorithm is proposed in this paper for crack detection. Based on the advantages of YOLOX-x, we add a semantic enhancement module to better use contextual information. The experimental results show that the anchor-free algorithm performs slightly better than other algorithms in crack detection situations. In addition, the proposed method displays better detection performance for slender and inconspicuous cracks, with an average precision of 0.858.

## Introduction

As the service lives of metros grow, various defects will inevitably appear in metro tunnels. Studies have shown that the presence of cracks increases the likelihood of other types of damage in tunnels, such as water leakage. Therefore, the detection of tunnel cracks is essential for ensuring metro safety.

To efficiently detect cracks, most existing methods utilize deep learning and surface information obtained from images of high-definition industrial cameras. Wang et al.^1^, Xi Taiyue’s team^2^ established a CCD industrial camera tunnel image acquisition system to collect crack images respectively. Wu et al.^3^ developed a CMOS line array camera-based rapid detection system for imaging metro tunnel cracks. Wang et al.^4^ proposed a deep and multiscale network (CrackNet-M) for detecting small or thin asphalt pavement cracks, the experimental results demonstrate that CrackNet-M can effectively detect both thick and thin cracks from various pavement surfaces with a high level of Precision (94.28%), Recall (93.89%), and F-measure (94.04%). Zhang et al.^5^ propose an encoder-decoder network (EDNet) for crack segmentation to overcome thequantity imbalance between crack and non-crack pixels, which causes many false-negative errors. Considering the cost, the number of cameras installed in most of the devices is limited, and the diameter of most metro tunnels is in the 6–8 m range, then the images will have a large field of view and the proportion of pixels occupied by the cracks in the images is very small. In addition, the lack of light and disturbances (such as cobwebs and pipes) in metro tunnels make it more difficult to detect small cracks. To address this issue, Wang et al.^6^ at Beijing Jiaotong University proposed a method to divide a global image into a few pieces and utilize a local image processing method to reduce the missed detection rate for small cracks. In this method, crack images are preprocessed, and features in the grid area are extracted, effectively avoiding the crack recognition interference associated with the complex tunnel lining background and insufficient light. The crack recognition rate of this method reaches 84%, but this method requires image preprocessing, cropping the images and splicing the subsequent recognition results, which increases the workload.

Regarding deep learning detection methods, anchor-based algorithms are the mainstream methods for crack detection, such as the R-CNN series, SSD, and YOLOv2-v5. Liu^7^ proposed an improved DeepLabV3+ network with 74.11% crack detection accuracy using CCD pictures. Dawei et al.^8^ proposed a multilayer feature fusion network based on Faster R-CNN, which detects cracks after preprocessing the captured tunnel surface images with image contrast enhancement and stitching methods. Fang^9^ used the improved YOLO v5 target detection model for metro tunnel identification, obtaining an AP value of 77.7% for cracks. Xue^10^ used CCD high-definition images based on the Faster R-CNN deep learning framework combined with the VGG-16 network and K-means clustering algorithm, obtaining a crack detection accuracy of 77.28%. Although the methods mentioned above can identify defects, these detection algorithms based on an anchor frame are best for large-target detection and often neglect small cracks. In addition, anchor-based models have difficulty balancing the recall of small targets and the computational cost. This easily leads to an extreme imbalance between the positive samples of small targets and large targets^11^. Because most anchor sizes and aspect ratios are present, the models are not universally applicable for all kinds of targets, which leads to poor performance for handling targets in cases in which the aspect ratio varies greatly, such as for cracks. Therefore, obtaining a better recognition algorithm adapted to the characteristics of metro tunnel cracks, especially small cracks, is important.

Many researchers have investigated anchor-free algorithms, and there are two main types of anchor-free detection frames as follows: (1) Algorithms based on key points, which detect the upper-left and lower-right corner points of the target first and then form the detection frame by combining the corner points, including CornerNet, CenterNet, ExtremeNet, etc., and (2) Center-based detection algorithms, which directly detect the center region and boundary information for the targets, including FCOS, CenterNet-TTFNet, etc.

In this paper, regarding the problem that the low percentage of crack areas in the images leads to poor recognition performance, we investigated how to better recognize small cracks without increasing the effort of image cropping and stitching.

- A self-developed tunnel image acquisition system was used to realize the rapid and nondestructive acquisition of tunnel surface information;
- Crack analysis and dataset construction were performed. Due to cracks often being long and thin and the existence of many forms of linear interference on the tunnel surface, crack textures and interference textures were labeled. We analyzed each crack in relation to neighboring objects to construct contextual information;
- Experiments were conducted regarding the performance of anchor-free algorithms and anchor-based algorithms in crack identification;
- To improve the recognition performance of algorithms for small cracks without the fine-scale segmentation of CCD images, we proposed a novel feature fusion network based on the structure of YOLOX-x. Experiments demonstrated that our method outperforms state-of-the-art methods in terms of small crack detection.

## Methods

### Data collection

**Figure 1:**
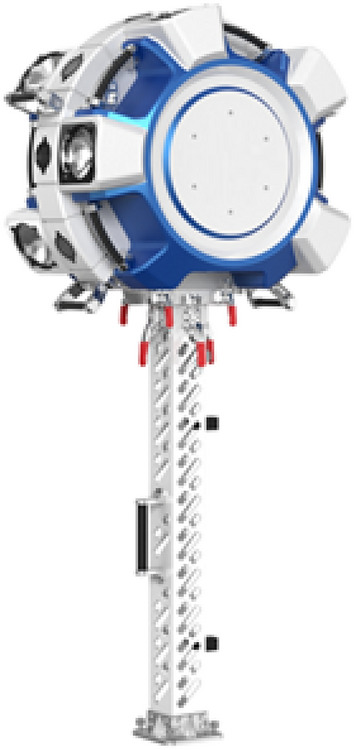
Tunnel surface information detection subunit.

In this paper, a self-developed tunnel image acquisition system is used to efficiently obtain information. As shown in Fig. 1, the system utilizes eight sets of industrial cameras and flashes and integrates a supporting industrial computer, a mileage measurement module, a synchronization control module, etc.

The specific parameters and indexes of the industrial cameras are shown in the following Table 1.

**Table 1:** Parameters of equipment.

Camera modules	Parameters	Parameter value
Light-sensitive chip	Light-sensitive chip	IMX267
	Shutters	GlobalShutter
	Size of the target surface	1″
	Light sensitive chip type	CMOS
	Size of light sensitive chip	14.1 mm × 7.5 mm
	Horizontal/vertical resolution	4096 px × 2168 px
	resolution (of a photo)	9MP
	Horizontal/vertical pixel size	3.45 µm × 3.45 µm
	frame rate	32fps
EMVA’ data	Quantum efficiency (typical)	68.0%
	Dark noise (typical)	2.3e^−^
	Saturation capacity (typical)	10.3ke^−^
	Dynamic range (typical)	72.8 dB
	Signal-to-noise ratio (typical)	40.1 dB
Camera's data	Interface	USB3.0
	Pixel bit depth	10or12bits
	Synchronization	Software trigger; free-run; hardware trigger
	Exposure control	Hardware trigger; programmable via the camera AP I
	Digital inputs	1
	Digital outputs	1
	General purpose I/O	2
	Power requirements	Power requirements
	Power (typical)	3W
Design	Design	Box
	Housing dimensions (L × W × H)	35.8 mm × 40 mm × 30 mm

### Metro tunnel crack properties

**Figure 2:**
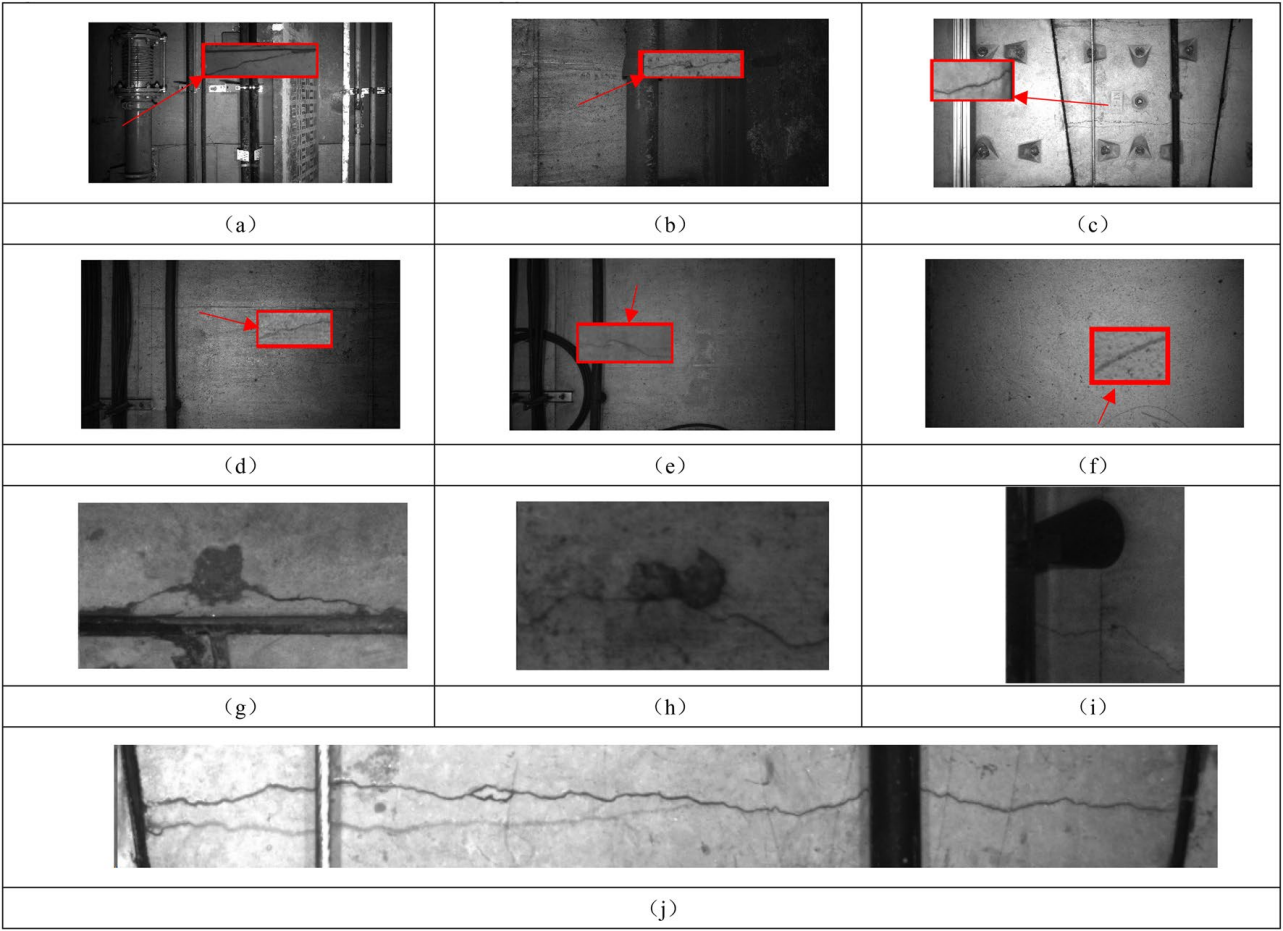
Example of crack.

The large Scale of images, the lack of light in underground tunnels and the presence of disturbances on the tunnel surface lead to poor crack identification^12^. In this paper, we analyze the information contained in the images acquired by a tunnel camera system. Then, we determine the interference factors disrupting crack recognition and construct datasets including real crack textures, interference noise textures, and background information for cracks. As shown in Fig. 2, the size of these images is 4096 px × 2168 px.

The surface cracks are shown in Fig. 2a–c, and the interference noise texture is shown in Fig. 2d–f. To display the crack information clearly, the details of the cracks are enlarged and displayed in the embedded images outlined in red boxes in Fig. 2a–f. More details are shown in Fig. 2g–j.

**Figure 3:**
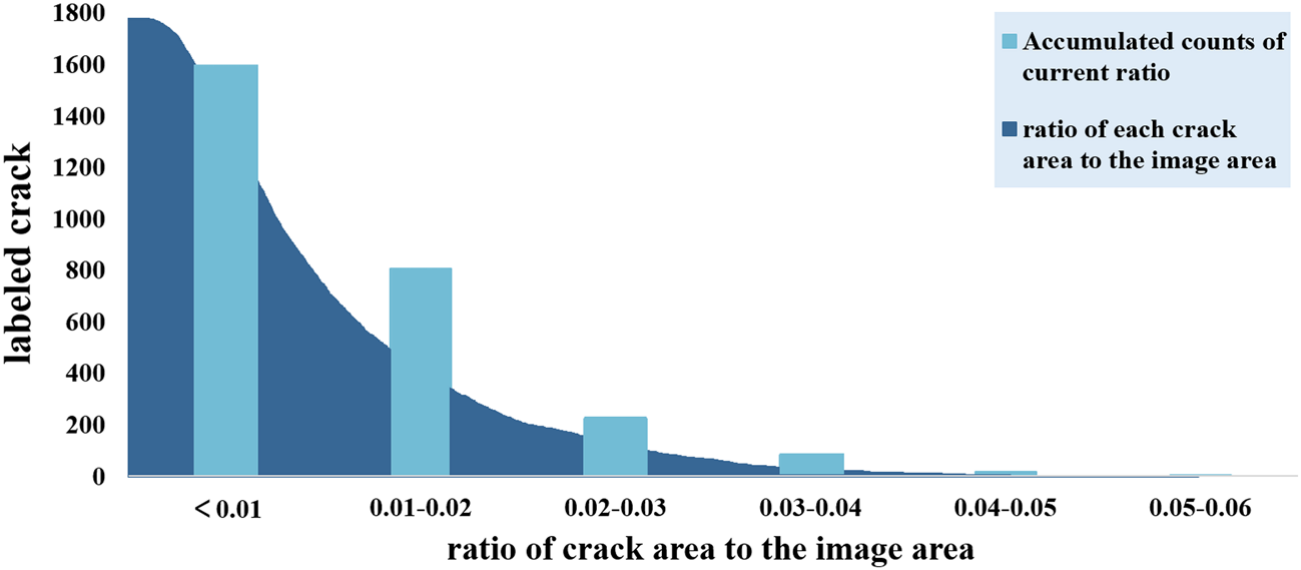
The ratio of the crack area to the image area.

In Fig. 3, the MASK files are generated when labeling the cracks with Labelme, the pixels of cracks in the MASK files can be calculated to obtain the area of each crack as *a*. With the size of each image fixed at 4098 px × 2048 px, we calculate the area as *b*. Divide a by b to get the area ratio. The ratios are generally less than 6%. Therefore, cracks are defined as small targets in this paper.

**Figure 4:**
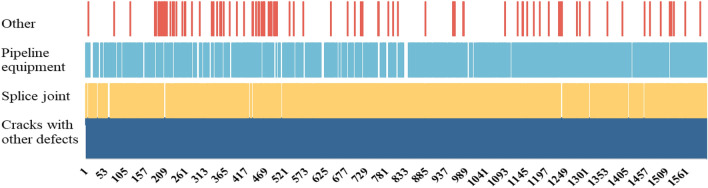
Statistical chart of crack labeling features.

According to the above crack characteristics, corresponding sample labeling datasets, which include crack samples, interference texture samples, and crack peripheral feature information samples (including splice joint information, pipeline equipment information, water seepage, and fallen concrete blocks), are established in this paper. According to the information in the images, water seepage and fallen lining blocks have similar texture characteristics, so they are grouped into the same class. The quantitative statistics for the labeled sample information are shown in Fig. 4.

Figure 4 depicts some of the crack data from the labeled sample, with 1600 records in total. The vertical axis indicates the characteristics of each crack. Based on the large amount of collected image data, the following characteristics are present in most of the cracks: (1) The cracks present a fine linear shape, and since their width values are usually ≤ 3 mm, the proportion of pixels occupied by the cracks is very small in the images obtained with the existing acquisition tools (no more than 6% of the area ratio); therefore, in this paper, cracks are treated as small targets. (2) Not all cracks appear exactly the same, exhibiting large differences in color, width, depth, and shape. Simultaneously, many interference factors exist on the surface of the tunnel walls, including cobwebs and water stains. The shapes and colors of these objects are similar to those of the cracks, greatly impacting the identification and prediction performance of the model. (3) Most cracks are accompanied by obvious features, as shown in Fig. 2 (7–10). Cracks are likely to occur in the parts of the tunnel wall where the shape of the material changes, such as the points at which the pipeline is affixed and the seams of ring pieces, and these cracks are obscured or truncated. (4) Some cracks are accompanied by water leakage and falling blocks, as shown in Fig. 2 (7–8). Existing studies have also shown that the emergence of cracks can lead to water seepage and falling block defects to a certain degree, which is consistent with the observations in this paper.

Therefore, the above information is considered the reference information for target detection, providing the model with background information regarding the presence of objects around the cracks and allowing the model to obtain comprehensive contextual features to further improve the confidence of crack detection.

### Discussion on the performance of anchor-free and anchor-based algorithms in crack recognition

The existing anchor-based detectors are divided into two main types: two-stage algorithms and single-stage algorithms. In this paper, representative and efficient detection algorithms are chosen for comparison, including the two-stage algorithms Faster R-CNN^13^ and MASK R-CNN^14,15^ and the single-stage algorithm YOLOv5. Considering that the cracks exhibit narrow and long linear characteristics, to achieve optimal detection performance, a clustering analysis is used before training to determine an optimal set of anchor sizes; notably, we use the K-means algorithm combined with the genetic algorithm to cluster the ground-truth boxes of the cracks for all the crack samples. The results show that the set of suitable anchor box aspect ratios is 0.27, 4.0, and 5.0 for MASK R-CNN and Faster R-CNN. The YOLOv5 network structure has a module to calculate the best recall of the labeled information in this dataset for the default anchors to obtain suitable anchor box aspect ratios, therefore, no adjustments are made to YOLO-v5 in this paper.

CornerNet^16^ is a classic anchor-free algorithm based on key points. Since CornerNet was proposed by Hei Law in 2018, anchor-free algorithms have been developed rapidly, allowing the target detection problem to be converted into a key-point detection problem and providing new ideas for target detection research. FCOS^17^ is a center-based classic detection algorithm proposed by Zhi Tian in 2019 and tested on the COCO2017 dataset with an AP_50_ value of 57.5. In 2021, Ge et al.^18^ proposed YOLO-x, which draws on the anchor-free fundamentals of FCOS, is faster and provides better performance. In this paper, the classic and efficient anchor-free detection algorithms CornerNet, FCOS, and YOLO-x are used to detect cracks. The experimental setup is shown in Table 2.

**Table 2:** Anchor-based and anchor-free algorithms.

Models	Release year	Anchor
Faster R-CNN (modified)	2016	Anchor-based
MASK R-CNN (modified)	2017	Anchor-based
YOLO v5	2020	Anchor-based
CornerNet	2019	Anchor-free
FCOS	2019	Anchor-free
YOLOX-x	2021	Anchor-free
YOLO v8	2023	Anchor-free

The above models are used to identify cracks, using AP50 for evaluation and the results are shown in Table 3.

**Table 3:** Comparison of experimental results.

Models	YOLO v5	Faster R-CNN (modified)	MASK R-CNN (modified)	CornerNet	FCOS	YOLOX-x	YOLO v8
AP 50	0.202	0.419	0.445	0.439	0.480	0.836	0.556
FPS	38.462	6.636	7.752	30.165	17.536	27.056	142.875

For the detection results of the crack, most anchor-free algorithms perform better in FPS (Frames Per Second) and the AP value. YOLOX-x shows a significantly high accuracy among all models while maintaining a good FPS value, reaching an AP value of 0.836. In this paper, the detection accuracy is mainly considered, and the YOLOX-x framework will be further optimized according to the crack characteristics to improve the detection performance of small cracks.

### Optimized YOLOX-x based crack detection network

Anchor-free detectors are developing rapidly. Researches^19–21^ have shown that an anchor-free mechanism significantly reduces the number of anchor parameters and the work of many anchor mechanisms, simplifying the detector and negating the need to set the size of the fixed anchor boxes in advance, which also makes this mechanism more suitable for small target detection.

YOLO-x uses “Decoupled Head”, “Data Aug”, “Anchor Free” and “SimOTA Sample Matching” to build an anchor-free end-to-end target detection framework and achieve first-class detection. The YOLO-x algorithm transforms the YOLO-x model, depending on the width and height of each network, into a variety of optional networks with standard or lightweight network structures. The standard network structures^22^ include YOLOX-s, YOLOX-m, YOLOX-l, YOLOX-x, and YOLOX-Darknet53. The lightweight network structures include YOLOX-Nano and YOLOX-Tiny. The AP values obtained from YOLOX-x testing based on the COCO dataset reveal that the YOLOX-x version yields the highest accuracy, with an AP value of 51.2%^23^. Therefore, the network detection algorithm proposed in this paper is an improvement based on the YOLOX-x structure.

**Figure 5:**
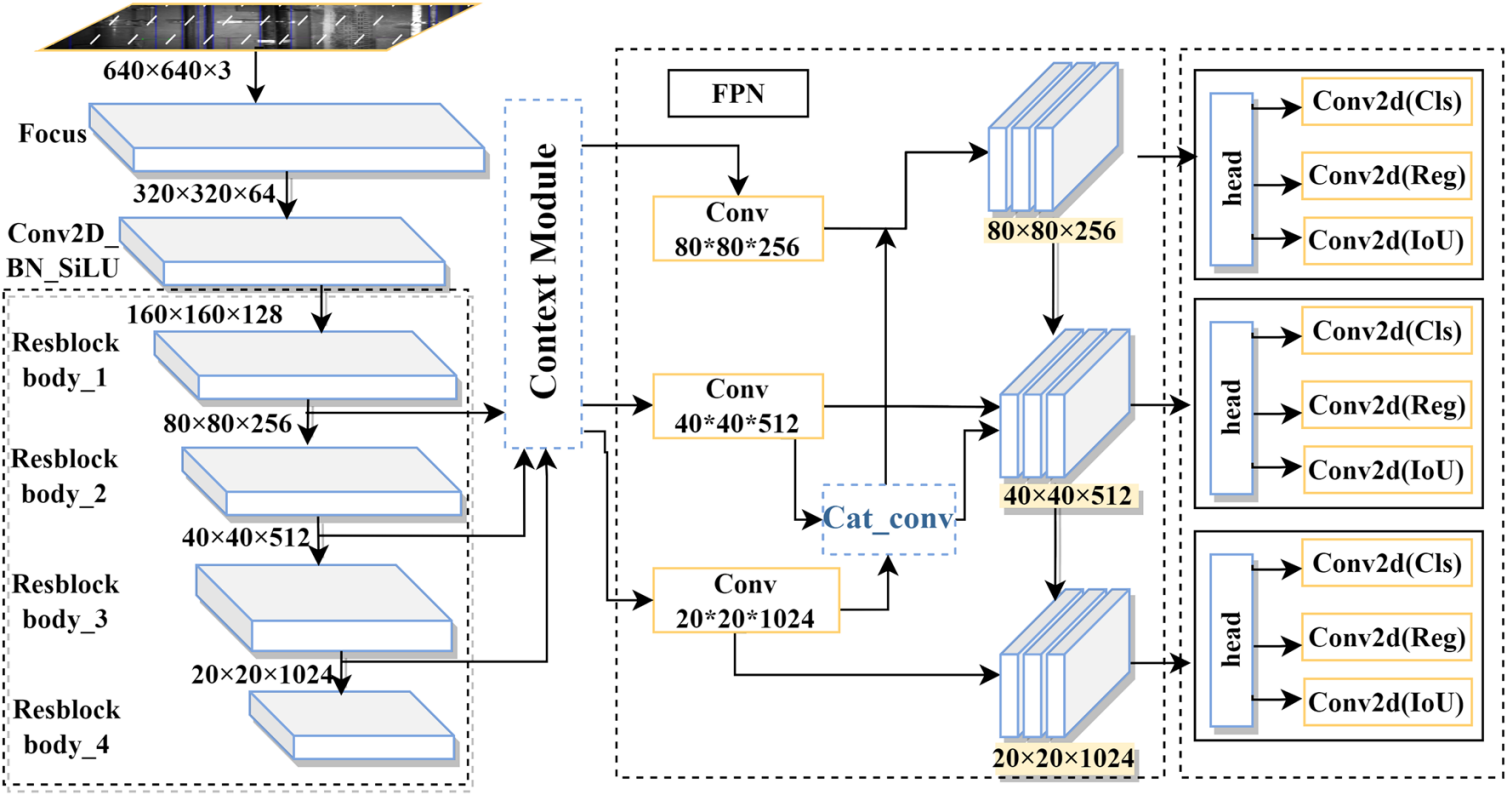
Network structure.

The method proposed in this paper is based on the anchor-free algorithm of YOLOX-x, which uses the CSPDarknet53 network as the backbone and performs feature extraction on the input image using ResBlock body_1–4. Relying on multi-scale feature fusion, three feature layers with sizes of 80 × 80 × 256, 40 × 40 × 512, and 20 × 20 × 1024 are enhanced by the context module, which allows the network to pay more attention to the contextual information of the cracks and better learn the information at each scale. Then, a path aggregation network (PANet) is used to extract deep features from the three feature layers and finally pass this information to the three decoupled heads, where the anchor-free method is used to predict the targets. The methodology used in this paper is shown in Fig. 5.

To fully use the hierarchical features of different feature layers and improve the model's understanding of the relationship between cracks and the surrounding environment, the relationship between cracks and the acquired contextual information (suspected seepage areas, fallen block areas, splice joints, etc.) is modeled by introducing a context module in the feature layer to increase the confidence value of small crack detection. Since the high-level feature layers have more semantic and global contextual information, and the bottom-level feature layers contain detail information, this paper relies on the multi-scale feature fusion for feature extraction and fusion from different feature layers. FPN^24^ mitigates the information diffusion problem by horizontally fusing the low-resolution feature layers with the high-resolution feature layers. However, a direct fusion of information with different densities causes semantic conflicts, which limits the expression of multiscale features and makes tiny targets easy to drown in the conflicting information. Therefore, in this paper, we adopt the Context module to obtain the context information of different feature layers by dilation convolution with different dilation rates and input them into FPN from top to bottom to enrich the context information.

**Figure 6:**
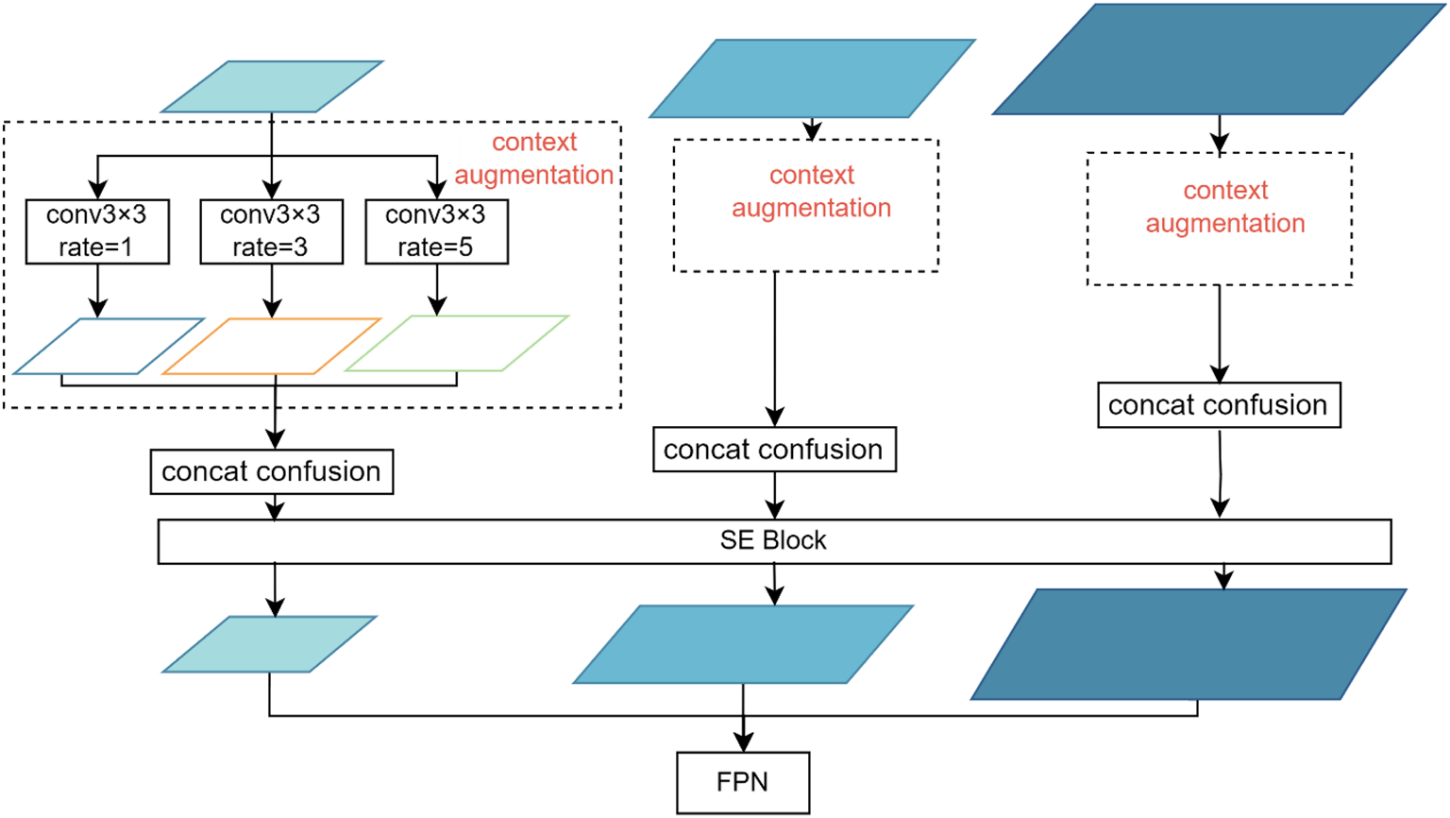
Context module.

The context module structure is shown in Fig. 6. The structure consists of context augmentation, a compression excitation block [squeeze-excitation block (SE block)], and feature fusion by concatenation.

Dilation convolution with different expansion rates is used to obtain context information for different receptive fields, and the FPN is applied from top to bottom to enrich the context information. Before the FPN, for all feature layers, hollow convolution with dilation rates of 1, 3, and 5 is performed through the context augmentation module to obtain semantic information for different sensory fields without increasing the number of parameters.

*SE Module* After the feature extraction part of the model, three feature layers with sizes of 80 × 80 × 256, 40 × 40 × 512, and 20 × 20 × 1024 are obtained, with the shallow layer providing a higher resolution and the last feature layer providing stronger semantic information than the other layers. To reasonably utilize the advantages of all feature layers, most algorithms use a feature pyramid network (FPN) to fuse the shallow and deep information, aiming to fully use the features of each layer and accurately identify defect areas. However, due to the unreliability of region details in some layers and information loss during down-sampling, when using FPN to detect objects under certain conditions (e.g., when objects are small, occluded, or truncated), directly fusing information with different densities can cause semantic conflicts. This limits multiscale feature expression and degrades the accuracy of small-target detection.

Existing studies^25–27^ have shown that integrating a learning mechanism into a network helps capture the spatial correlations between features, and by assigning different weights to different locations in an image from the perspective of the channel domain, important feature information can be obtained. The squeeze-and-excitation attention mechanism, which is a method of determining weights in the channel-attention paradigm, is used to model network convolution by explicitly modeling the interdependence among feature channels and obtaining the importance of each feature channel. By explicitly modeling the interdependence among feature channels, the importance of each feature channel is obtained. In accordance with these results, useful features are promoted, and the features that are less useful for the current task are suppressed to assign weights among different channels and obtain the primary and secondary priorities. Through the network structure, making the size of the feature map from size (N, C, H, W) to (N, C, 1, 1) to fuse global context information, where “N” represents the batch size, “C” represents the number of channels of the feature map, “H” represents the height of the feature map, and “W” represents the width of the feature map. Then, the excitation operation is used to consider the model complexity based on the nonlinearity of the fully connected layers to determine the weights of different channels. Then, the reshape-over weight values are multiplied by the original feature map to obtain feature maps with different weights. The SE block is simple in structure and supports lightweight computations, so it only slightly increases the model complexity and computational burden, and it can be added anywhere in the YOLO-x network.

The location of the SE module should be carefully selected. The module is generally added at the bottom of the backbone network, anywhere in the FPN, or between layers in the backbone network. Therefore, in this paper, the SE module is added (a) before the FPN and after the backbone, (b) between the shallow and deep layers of the FPN when the concatenation operation is performed, and (c) at the end of the FPN. Tests show that the accuracy is 0.84 after adding the module at (a), 0.78 after adding it at (b), and 0.63 after adding it at (c); therefore, context augmentation is used to augment the information for the three feature layers corresponding to the output of the 256-, 512-, and 1024-dimensional channels for the FPN output from the backbone network.

Possible fusion methods include the addition-based fusion method, adaptive fusion method, and concatenation fusion method. Research has shown that the concatenation method exhibits the largest improvement for small targets, the adaptive fusion method yields the largest improvement for medium and large targets, and the improvement achieved through addition-based fusion method is comparatively balanced. Therefore, in this paper, the Concat-fusion method is used to obtain spatial adaptive weights based on convolutional cascading and SoftMax operations.

The concatenation operation is used for fusing multiscale dilation convolution features to obtain rich contextual information for feature enhancement. The concatenation method is shown in Eq. 1:

1 $$Z_{concat} = \mathop \sum \limits_{i = 1}^{c} X_{i} *K_{i} + \mathop \sum \limits_{i = 1}^{c} Y_{i} *K_{i + c}$$

Each feature layer corresponds to a matrix, and a larger feature matrix is generated by concatenating multiple feature matrices along the specified dimensions, where ${Z}_{concat}$ denotes the output result after fusion, ${X}_{i}$ and ${Y}_{i}$ denote the channels of the $i\_th$ input to feature layers X and Y, respectively, ${K}_{i}$ and ${K}_{i+c}$ are the corresponding weight matrices, and $c$ denotes the number of channels of the feature. The contextual information can be aggregated to the output by calculating the weighted sum.

**Figure 7:**
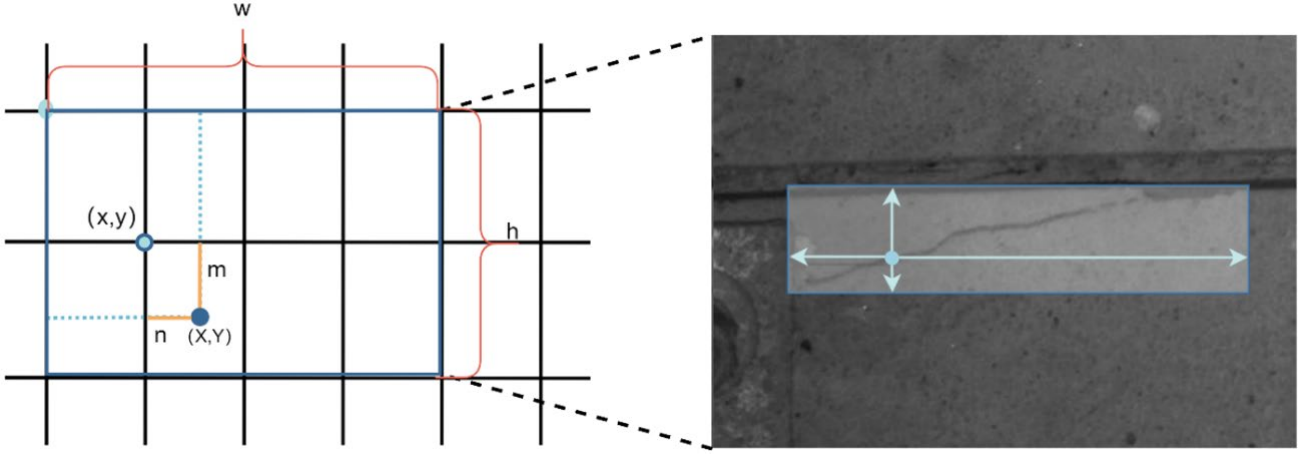
Anchor-free mechanism.

*Anchor-Free Detectors* Most anchor-based target algorithms^28–30^ generate multiple rectangular anchor boxes for each pixel point through a set of aspect ratios and calculate the degree of coverage between the anchor boxes and the ground truth boxes to select the appropriate anchor boxes as the final prediction boxes; these anchor boxes are not universal for all targets. In this paper, crack detection using the anchor-free method is performed, as shown in Fig. 7. This method is based on the principle that in the prediction step of model classification, the four parameters of prediction boxes, i.e., the coordinates of the upper-left corner and the values of the width and the height of the prediction box, are directly generated for each pixel point. These values are then mapped back to the original image.

In Fig. 7, the parameters (m, n) correspond to the offset of the center point, (X, Y), of the predicted target relative to the upper-left corner of the grid cell (x, y), and w and h denote the width and height of the target. These values vary based on the scale of the relative predicted feature maps, which are then mapped from the current feature layer back to the original image.

Suppose that for a point on the predicted feature map mapped back to the original image, the coordinates are ($${O}_{x}$$, $${O}_{y}),$$ and the step size of the feature map with respect to the original image is s. In this case, the network predicts that the coordinates of the target bounding box corresponding to this point are as follows:

2 $$\left\{ {\begin{array}{*{20}c} {O_{x} = \left( {X + n} \right)*s } \\ {O_{y} = \left( {Y + m} \right)*s} \\ \end{array} } \right.\left\{ {\begin{array}{*{20}c} {W = e^{w} *s } \\ {H = e^{y} *s} \\ \end{array} } \right.$$

3 $$\left\{ {\begin{array}{*{20}c} {W = e^{w} *s } \\ {H = e^{y} *s} \\ \end{array} } \right.$$

## Experiments and results

Based on a total of 2750 images (4096 px × 2168 px) obtained by the image acquisition system, crack samples, interference texture samples, and crack background information samples (including seam information, pipeline equipment information, water seepage areas, and fallen block information) are labeled in the LabelMe environment. The numbers of training images and validation images are divided at a ratio of 8:2; the training set includes 2103 images, and the test set includes 647 images. Experiments are based on PyTorch and a GPU (12 G) environment, the training batch size is set to 8. After the model gradually converges, and the trained model is utilized for prediction.

To illustrate the effectiveness of the proposed improved algorithm, corresponding ablation experiments are conducted, and contextual information is added to the model, as shown in Table 4.

**Table 4:** Ablation experiments setup.

Model	YOLO v5	Modified faster R-CNN	Modified MASK R-CNN	YOLOX-x	YOLOX-x with SE	The method in this paper
No contextual information	0.202	0.419	0.445	0.836	–	–
Contains contextual information	0.693	0.468	0.394	0.817	0.862	0.874

The results in Table 4 show that the crack recognition accuracies of the YOLOv5, fixed Faster R-CNN and YOLOX-x series algorithms are improved, especially that of YOLO V5, which exhibits significantly enhanced crack recognition but still displays a lower accuracy than YOLO-x. Adding contextual information to the fixed Mask R-CNN instead decreases the detection accuracy; notably, the contextual information interferes with the recognition process of the model. Therefore, this method is not desirable for use with the Mask R-CNN model. After adding the SE module, the accuracy of YOLO-x increases to 0.839, and with the proposed method, i.e., using the context augmentation module and the contextual information from YOLOX-x, the AP value of the recognition of cracks reaches 0.874, indicating better detection results than those obtained with the original model.

In order to demonstrate the reliability of the method presented in this paper on the data set, a k-fold cross-validation is carried out. The dataset is divided into k equally sized image subsets. These k subsets are traversed sequentially, using the current subset as the validation set and all remaining images as the training set for training. Finally, the average of the k evaluation metrics is taken as the final evaluation metric. Here k is taken as 5. The AP values of the traversals are 0.795, 0.916, 0.799, 0.907, 0.874 and the average is 0.8582. The variance of the cross-validation results is 0.00269, which shows that the overall fluctuation of the AP values is small and the model is stable.

## Discussion

**Figure 8:**
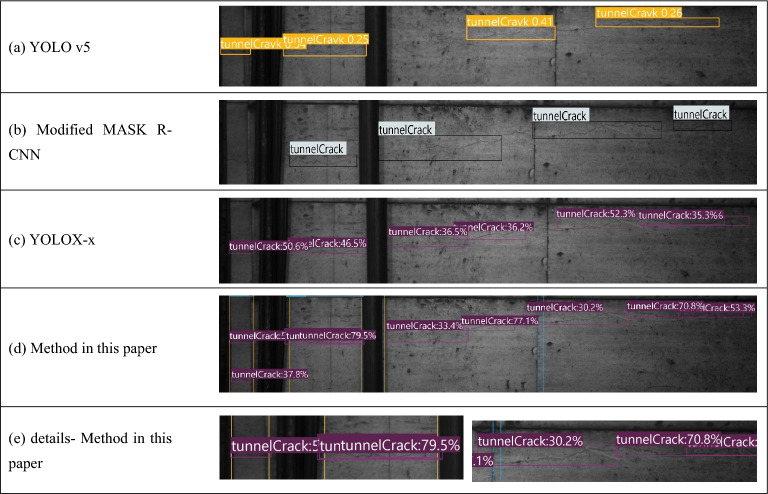
Predicted perceptual effects.

The prediction results obtained from the representative models in Table 4 are selected for analysis. Figure 8a,b shows the model prediction results. The original image contains a long crack and a short crack. The long crack is distributed around the splicing joint and is divided into three segments by two pieces of pipeline equipment. The identification results show that all four models can effectively predict and localize long cracks, but differences in identification ability are clear.

The result of YOLO v5, depicted in Fig. 8a, has more omissions than the other results. The long crack is not fully identified (the crack on the right side of the pipeline equipment and on the right side of the splicing seam), and a less obvious crack with narrow and tiny features below the long crack is missed. The Mask R-CNN result, depicted in Fig. 8b, also has omissions. Notably, the model misses a crack that is split into two parts by the pipeline equipment, fails to recognize the leftmost part of the long crack, and similarly fails to detect the tiny crack below the long crack. The YOLOX-x model in Fig. 8c, which identifies the long crack relatively completely, similarly omits the tiny crack below the long crack. In Fig. 8d, the tiny and shorter crack below the long crack is detected by optimize YOLOX-x, which means a few missed detections are avoided, and better results are obtained. Notably in Fig. 8e, the details of the detection results of the long cracks from Fig. 8d, show that the method in this paper can detect defect regions in terms of small crack detection, but the quality of the regression boxes is poor, even we used the NMS, as demonstrated by the fact that multiple duplicate prediction boxes are formed in the detection of the same crack in some cases. This situation occurs frequently in this experiment, which is a drawback of the method.

Combining the results of Tables 3 and 4 shows that the anchor-free algorithms are effective for crack detection and outperform the anchor-based algorithms in this experiment. YOLOX-x exhibits both high performance and high speed. In this paper, two factors are considered responsible for this phenomenon. First, the short training time of YOLOX-x is related to its network structure and to the fact that it is an algorithmic structure without anchor frames, reducing the number of parameters related to the anchor frames. Both of these characteristics reduce the computational effort of the model. In addition, the target cracks in this paper present linear characteristics of varying lengths and are extremely thin and narrow. The universality of anchor-free networks in target detection is well represented, and anchor-based algorithms somewhat affect crack detection by fixing the size of the anchor frames and predicting the width-to-height ratio of the anchor frames.

In conclusion, the model proposed in this paper accurately identifies cracks without generating misjudgments, avoids the interference caused by other textures on the surface of tunnel walls and is effective in detecting fine crack targets as well as cracks that are truncated by pipeline equipment.

## Conclusions

In this paper, the low percentage of crack areas in images, which leads to poor recognition performance, is explored, and we investigate how to better recognize small cracks without increasing the effort of image cropping and stitching. Based on a CCD high-definition camera in a self-developed tunnel image acquisition system, crack feature information is collected, analyzed and counted. A dataset is produced based on this analysis. Additionally, the crack detection performance of anchor-based and anchor-free algorithms is explored for thin and narrow cracks. Then, an improved YOLOX-x target detection algorithm is proposed, which utilizes the acquired context information (suspected seepage areas, fallen block areas, splicing joints, etc.) by introducing context enhancement and attention mechanisms to improve accuracy. The following conclusions are drawn: (1) Most of the cracks exhibit a thin, narrow and long linear shape, occupying a very small proportion of pixels in the CCD high-definition industrial camera images (the ratio of crack area to image area is 6% or less), and the cracks are prone to occur near the tunnel wall splice joints and points where pipes are joined, making the cracks appear blocked or truncated. Furthermore, the appearance of cracks is associated with areas of water seepage or fallen blocks to a certain extent. (2) Although anchor-free algorithms were developed after anchor-based algorithms, they provide similar or better detection results. And results show that unoptimized YOLOX-x has the advantages of both high performance and high speed, but it cannot accurately recognize small cracks in uncropped images (3) The proposed method exhibits better small crack identification performance, with an AP value of 0.858, and performances better small crack prediction results. However, the proposed algorithm still has deficiencies; notably, multiple duplicate prediction boxes are formed in the identification of the same crack in some cases.

### Supplementary Information

Supplementary Information.

## Data Availability

The datasets of this study are available from the corresponding author on reasonable request.
