# Supplementary material for: Effective small crack detection based on tunnel crack characteristics and an anchor-free convolutional neural network
Source: Sci Rep. 2024 May 6;14:10355. doi: 10.1038/s41598-024-60454-3 (PMC11074154; doi:10.1038/s41598-024-60454-3)
Supplement: Supplementary file 1 — Supplementary Information. [file 41598_2024_60454_MOESM1_ESM.pdf]

Effective small crack detection based on tunnel crack characteristics and an anchor-free convolutional neural network

Wang li[1], Tang Chao[2,*]

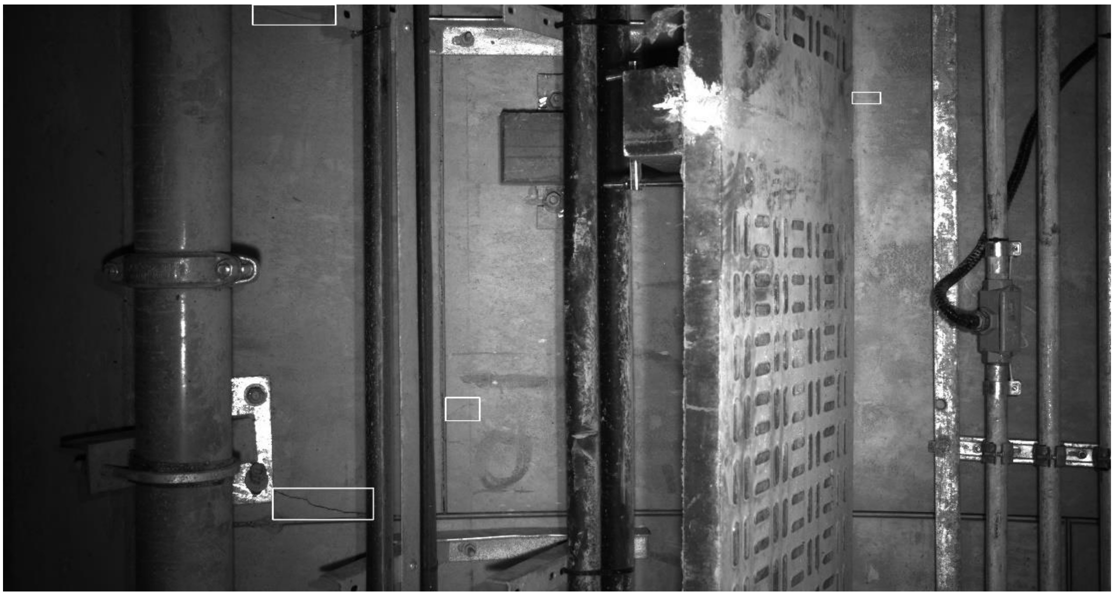

Supplementary Figure S1. An image from the dataset used in this paper

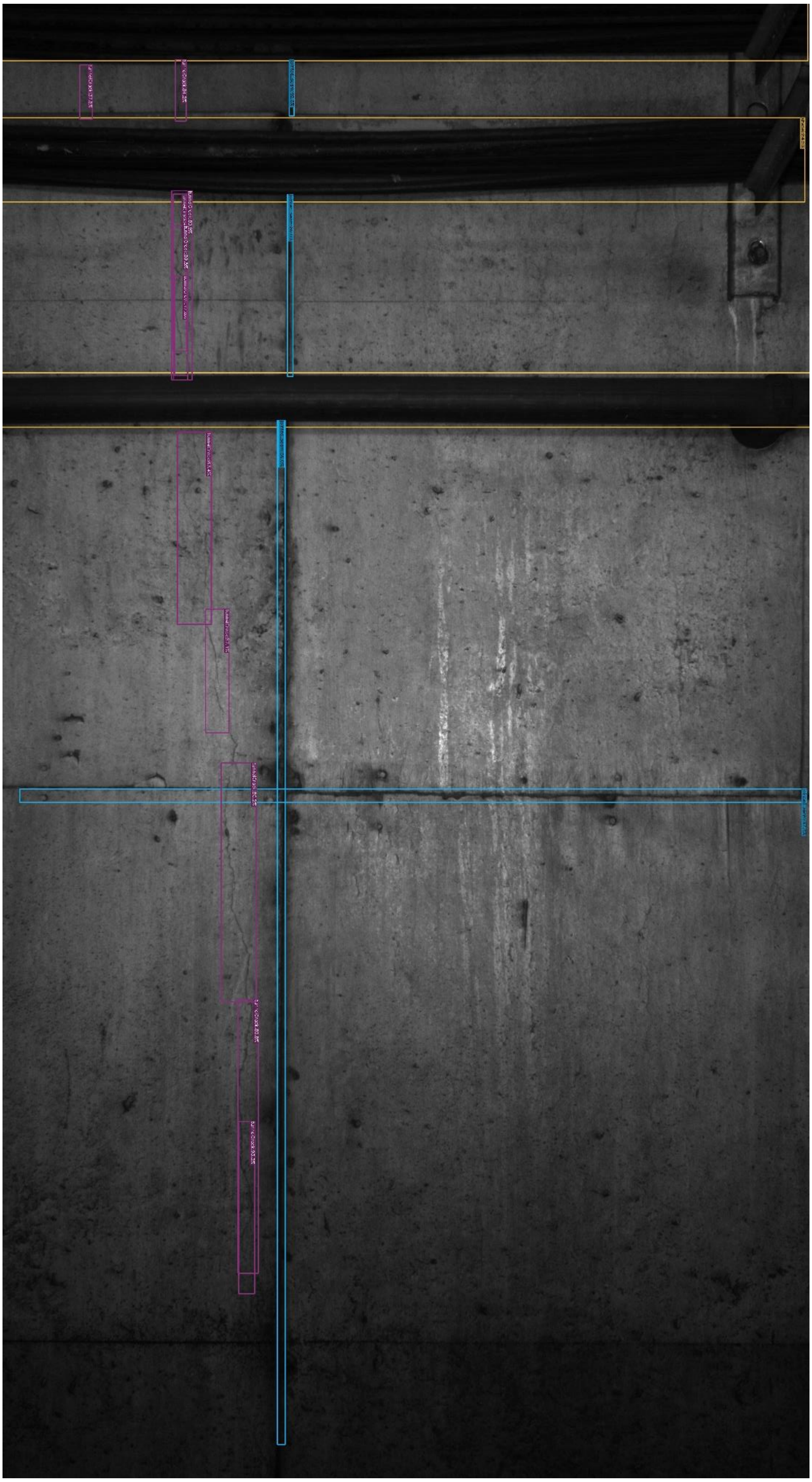

Supplementary Figure S2. Full size image of Figure9(5)Model6 in this paper
